# Successful Treatment of Adventitious Bursitis With Marginal Resection in a 23-Year-Old Patient: A Case Report

**DOI:** 10.7759/cureus.75615

**Published:** 2024-12-12

**Authors:** Şeyhmus Kavak, Arın Celayir, Ulas Yavuz, Firat Kargin, Ali Seker

**Affiliations:** 1 Department of Orthopedics and Traumatology, Cerrahpasa Faculty of Medicine, Istanbul University - Cerrahpasa, Istanbul, TUR; 2 Department of Orthopedics and Traumatology, Cerrahpasa Faculty of Medicine, Istanbul University - Cerrahpasa, ıstanbul, TUR; 3 Department of Pediatric Orthopedics, Cerrahpasa Faculty of Medicine, Istanbul University - Cerrahpasa, Istanbul, TUR

**Keywords:** adventitious bursitis, cartilage cap, humeral ostoechondroma, marginal resection, rapid growth

## Abstract

Adventitious bursitis is an inflammatory condition affecting bursae. Distinct from primary or infectious bursitis, adventitious bursitis typically develops secondary to conditions such as rheumatoid arthritis, gout, or repetitive joint overuse. The resulting inflammation can lead to pain, swelling, and restricted mobility, significantly impacting patients' quality of life.

In this paper, we present the case of a 23-year-old patient who reported to our clinic with swelling and pain localized to the right arm. Clinical examination, imaging studies, and histopathological analysis confirmed a preliminary diagnosis of adventitious bursitis associated with osteochondroma, which had exhibited rapid growth. Diagnostic evaluation included ultrasonography and MRI, which identified bursal inflammation and its relation to the underlying osteochondroma. The patient was treated successfully through marginal resection. Postoperative follow-up demonstrated significant symptom relief and restored joint functionality. This case underscores the importance of thorough diagnostic assessment and surgical management in achieving optimal outcomes for patients with adventitious bursitis.

## Introduction

Adventitious bursitis refers to the inflammation of a bursa, a fluid-filled sac that reduces friction between bones, tendons, and muscles around joints. Unlike primary or infectious bursitis, which results from trauma or bacterial infections, adventitious bursitis is often linked to conditions such as rheumatoid arthritis, gout, or repetitive joint overuse. Symptoms commonly include pain, swelling, and restricted joint mobility [1].

Accurate differentiation of adventitious bursitis from other forms, such as primary or infectious bursitis, is critical for proper diagnosis and treatment [2]. Effective management depends on understanding the underlying cause and tailoring treatment to address both symptoms and contributing factors [3].

Despite its clinical significance, there is limited literature detailing specific diagnostic challenges or surgical interventions, such as marginal resection, for adventitious bursitis. Most existing studies focus on conservative treatments or general management strategies without addressing cases complicated by structural abnormalities like osteochondromas. This case report contributes to the literature by highlighting the diagnostic complexities and surgical management of adventitious bursitis associated with a rapidly growing osteochondroma. The presented case underscores the importance of integrating imaging modalities and histopathological evaluations to ensure accurate diagnosis and optimal treatment outcomes, providing a valuable reference for clinicians managing similar cases.

## Case presentation

A 23-year-old female patient presented with complaints of swelling and pain in her right arm persisting for one month. The patient had a known diagnosis of osteochondroma in the right humeral shaft for the past 10 years. She sought medical attention due to a sudden increase in pain and the development of swelling associated with the osteochondroma. The patient had no other known medical conditions, was not taking regular medications, and had not undergone any previous surgical procedures. Informed consent was obtained from the patient before all medical interventions.

**Figure 1:**
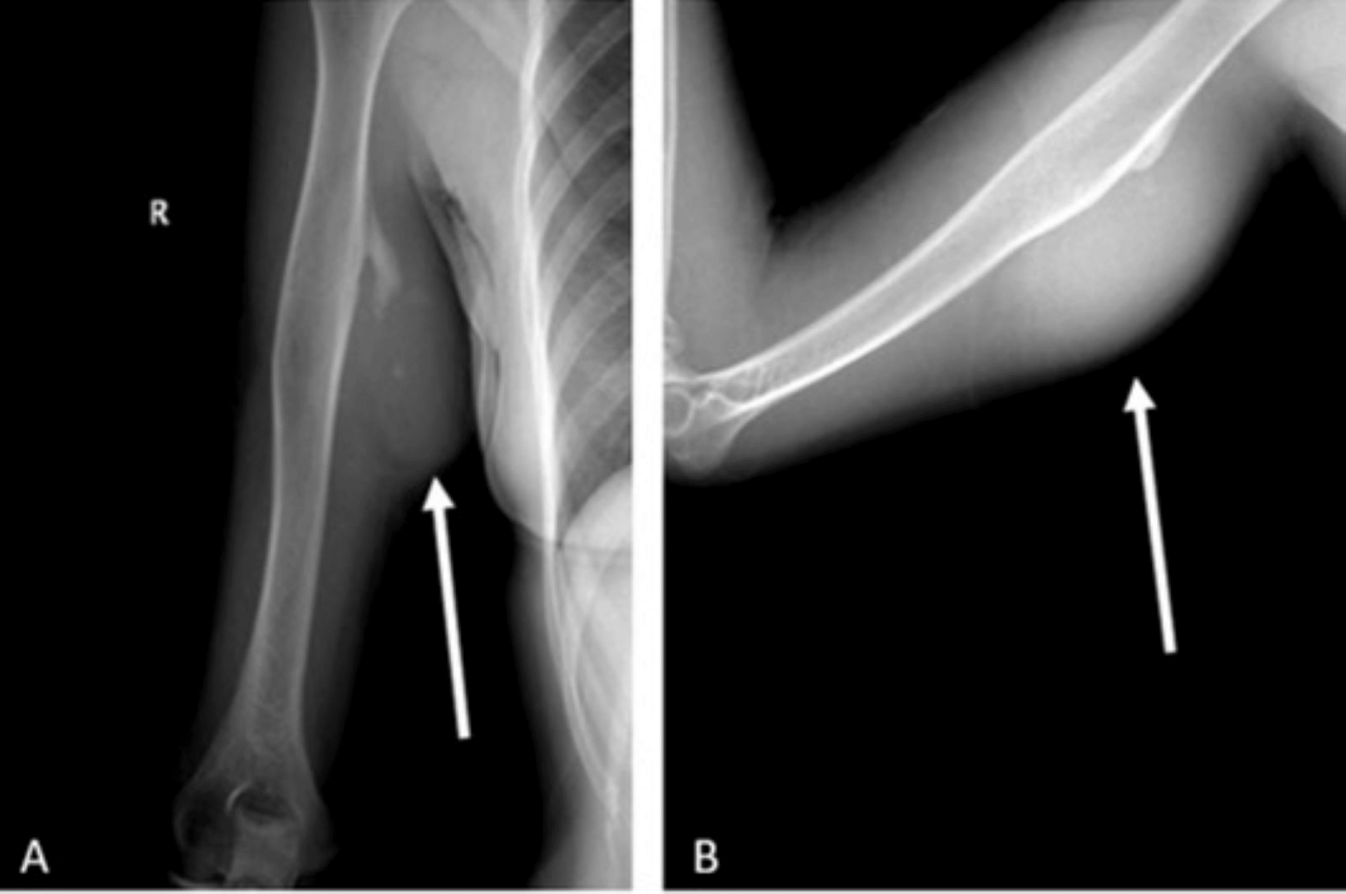
Preoperative X-ray images: (A) anteroposterior (AP) view of the humerus and (B) lateral view of the humerus. White arrows indicate the mass, which is located in the humeral shaft. Irregularities in both the bone and surrounding soft tissue are also observed.

**Figure 2:**
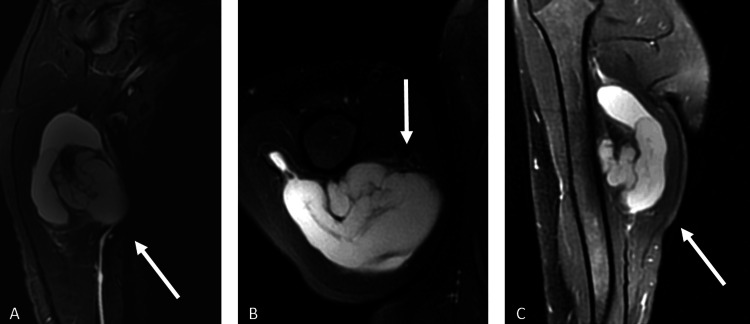
Figure 2: Preoperative magnetic resonance imaging (MRI) of the patient: (A) sagittal MRI view; (B) axial MRI view; and (C) coronal MRI view. White arrows indicate the lesion around the humerus.

Radiological assessments, including updated X-ray and MRI imaging (Figures 1-2), were performed at our hospital, confirming the preliminary diagnosis of osteochondroma. Following a multidisciplinary tumor board evaluation, which considered the patient’s symptoms, imaging findings, and potential risks of conservative versus surgical management, the decision was made to proceed with surgical intervention. Marginal resection was subsequently planned and successfully performed by our team.

**Figure 3:**
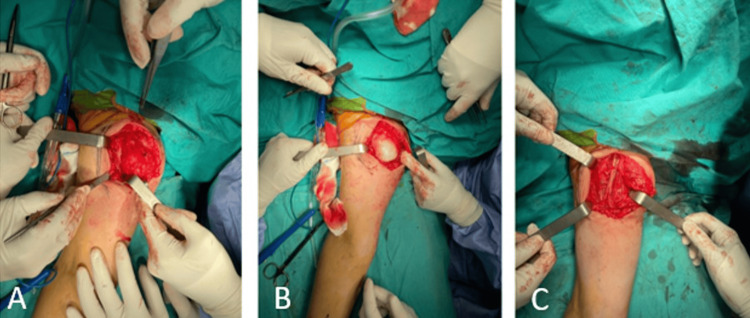
Intraoperative clinical images of the patient. The images show the lesion being surgically localized and excised. The proximity of the lesion to the axillary region is also observed.

A longitudinal incision measuring approximately 10 cm was made, extending from the medial-posterior aspect to the distal part of the right humeral shaft. The skin and subcutaneous tissues were incised, and the median nerve, brachial artery, and vein were identified and retracted laterally. Soft tissue dissection provided access to the bursa. Under fluoroscopic guidance, the lesion's location was identified. The osteochondroma was excised using an osteotomy from its pedicle, and the bursa was removed (Figure 3).

**Figure 4:**
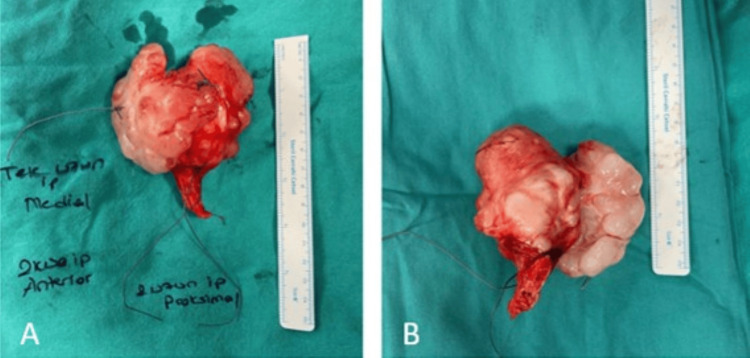
Intraoperative clinical images showing the site after removal of the lesion.

A specimen was separated for pathological examination (Figure 4).

**Figure 5:**
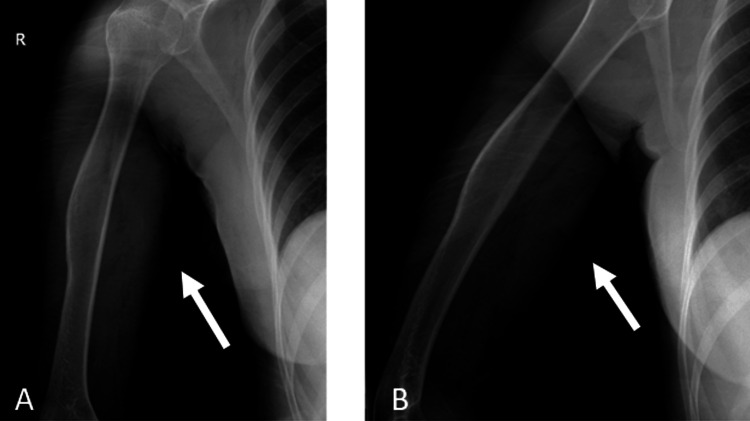
Postoperative X-ray images of the patient: (A) anteroposterior (AP) view of the humerus and (B) lateral view of the humerus. White arrows indicate the site of the lesion after removal.

The layers were closed using a Minivac drain following anatomical principles. Postoperatively, there was no discharge at the incision site. The patient's pain and swelling subsided after the surgery. She was discharged with a shoulder-arm sling one day postoperatively and scheduled for a follow-up in the outpatient clinic. At the six-month postoperative follow-up, no pathology was observed. The patient demonstrated a full joint range of motion and mobility, with no neurovascular deficits. Postoperative X-rays revealed no abnormalities (Figure 5).

## Discussion

Differentiating adventitious bursitis from other conditions is crucial for accurate diagnosis and tailored treatment. Potential differential diagnoses include primary bursitis caused by trauma or infections, such as bacterial infections. In our case, the patient had no trauma history, was diagnosed with osteochondroma a decade earlier, and showed no signs of infection. Both sedimentation rate and C-reactive protein levels were normal. The mass's rapid growth within a month raised suspicion of a tumor, but surgery confirmed adventitious bursitis.

Treatment of adventitious bursitis typically involves addressing the underlying cause and symptoms. For secondary conditions like rheumatoid arthritis or gout, managing the primary disease is essential. Nonsteroidal anti-inflammatory drugs (NSAIDs) are commonly used to relieve pain and reduce inflammation. Physical therapy can enhance joint flexibility and strength, while fluid aspiration or corticosteroid injections are options to alleviate symptoms. Rest and activity modification are often recommended. In severe or persistent cases, surgical removal of the inflamed bursa may be necessary. Surgical intervention, as performed in our case, aligns with current management guidelines, which recommend surgical resection in cases where conservative treatments fail, or complications such as rapid growth occur. Postoperative follow-up is critical to monitor for recurrence or complications such as infection, stiffness, or residual pain.

Adventitious bursitis affects individuals across all age groups and is often associated with repetitive movements, trauma, or underlying conditions such as rheumatoid arthritis and gout [4]. Our patient had no rheumatological conditions or excessive shoulder use. The condition was discovered incidentally and only prompted medical attention due to rapid growth and pain. Özdemir et al. discussed a case involving rapid proximal humerus growth with similar symptoms [5]. However, unlike their patient, ours had no trauma history, suggesting that rapid growth without trauma should also be considered.

To determine whether the rapid mass growth indicated malignancy, we used MRI imaging, which ruled out malignancy [6]. In another case by Poenaru et al., ultrasound was used alongside MRI for diagnosis [7]. While MRI sufficed in our case, incorporating ultrasound could strengthen the preliminary diagnosis. Current imaging guidelines for soft-tissue masses highlight the complementary role of ultrasound and MRI for improved diagnostic accuracy.

Although adventitious bursitis is more commonly reported in the knee and upper extremities, it has also been described in less common sites like the sole of the foot [8]. Our findings contribute to the growing recognition of adventitious bursitis in unusual presentations, particularly when associated with rapid mass growth. The lack of trauma or systemic conditions, in this case, emphasizes the importance of considering adventitious bursitis in differential diagnoses for atypical presentations. Long-term follow-up is essential to evaluate for recurrence and ensure complete resolution, given the potential for complications following surgical management.

## Conclusions

This case emphasizes the importance of distinguishing adventitious bursitis from other conditions to ensure accurate diagnosis and management. The rapid lesion growth without trauma or infection highlights the need for advanced imaging, such as MRI, to rule out malignancy. Successful surgical management, in this case, demonstrates the viability of marginal resection for atypical presentations of adventitious bursitis. Further studies are needed to better understand its pathogenesis, optimal treatment strategies, and long-term outcomes.
